# Non-traumatic Anorectal Foreign Bodies: A 10-Year Tertiary Center Experience

**DOI:** 10.7759/cureus.103822

**Published:** 2026-02-18

**Authors:** Subhi Mansour, Mohammad Radwan, Roy Abramov, Hayim Gilshtein

**Affiliations:** 1 Department of General Surgery, Rambam Medical Health Center, Haifa, ISR; 2 Department of General Surgery, Colorectal Surgery, Rambam Medical Health Center, Haifa, ISR; 3 Medicine, Technion - Israel Institute of Technology, Haifa, ISR

**Keywords:** anorectal foreign body, emergency surgery, examination under anesthesia, rectal trauma, sexual gratification

## Abstract

Background

Anorectal foreign bodies (AFBs) represent a challenging clinical problem with rising incidence, diverse presentations, and potential for serious complications. This study reviews a decade of experience at a tertiary referral center, analyzing demographics, management strategies, and outcomes.

Methods

A retrospective review was conducted of all patients presenting with AFBs between January 2014 and April 2024 at Rambam Health Care Campus. Data collected included demographics, type of object, diagnostic approach, management strategy, and complications. Statistical analyses used ANOVA, Chi-squared, and Fisher's exact tests, with p<0.05 considered significant.

Results

Thirty-nine patients were identified, 74.4% of whom were male. Males presented at an older mean age than females (49.4 ± 19.3 vs. 35.4 ± 12.5 years, p=0.015). Food products were the most common foreign body overall, and sex toys were reported equally among both genders (p=0.087). Almost half of the patients required surgical intervention (48.7%), while 28.2% were managed by manual extraction and 7.7% experienced spontaneous expulsion. Unspecified objects were significantly more likely to be managed non-operatively (p=0.016). Most cases were diagnosed clinically (71.8%), and 19 patients required surgery, including five who underwent laparotomy for proximal impaction or perforation. Postoperative complications were rare (2.6%), with no mortality observed.

Conclusion

Anorectal foreign bodies remain an uncommon but clinically important condition, predominantly affecting males, with significant gender differences in age but not in object type. While many cases can be managed conservatively, nearly half require surgical intervention, and a minority necessitate laparotomy. Complications were infrequent, suggesting that timely, structured management may contribute to favorable outcomes in experienced centers. Early recognition, standardized treatment algorithms, and stigma reduction may further improve outcomes and patient safety.

## Introduction

The incidence of anorectal foreign bodies (AFBs) presenting to emergency departments appears to be rising, representing an important and increasingly recognized form of anorectal trauma. AFBs may be introduced voluntarily or involuntarily, either by the patient or another individual [1]. Reported objects include household items such as bottles, glasses, light bulbs, and wooden rods, as well as sexual devices such as dildos and vibrators [2]. Reasons for insertion vary widely and include sexual practices, assault, psychiatric disorders, accidents, and self-treatment of constipation [2,3].

AFBs are most frequently observed in individuals aged 20 to 40 years, with a clear male predominance [3]. The true incidence is likely underestimated due to underreporting, as embarrassment and social stigma often deter patients from seeking timely medical attention. Presentations may be immediate or delayed, typically involving anal or abdominal pain, constipation, rectal bleeding, or fecal incontinence [4]. Accurate diagnosis relies on detailed history-taking, careful digital rectal examination, and, when indicated, adjunctive imaging studies.

Management of these cases remains challenging even for experienced clinicians, owing to delayed presentation, incomplete disclosure, and the diversity of foreign objects encountered. Complications such as bleeding, perforation, obstruction, and sepsis contribute to variable morbidity and occasional mortality [5-7]. Although most AFBs can be safely removed at the bedside or via transanal approaches, a subset of patients require operative intervention - often examination under anesthesia, and, in more severe cases, laparotomy for complications or failed extraction attempts [6,8].

The primary objective of this study was to describe clinical management patterns and outcomes of patients presenting with anorectal foreign bodies over a 10-year period. Secondary objectives included characterization of patient demographics, presentation patterns, diagnostic approaches, and factors associated with management strategies.

## Materials and methods

Study design

A retrospective, systematic review was conducted at Rambam Health Care Campus, evaluating emergency department records between January 1, 2014, and April 1, 2024. The study was designed to identify and analyze all cases of patients presenting with anorectal foreign bodies (AFBs) during this period. Because this was a retrospective review of all consecutive cases presenting over the defined 10-year period, no a priori sample size calculation was performed; all eligible patients were included to maximize completeness and representativeness. 

Patient recruitment

Patients were identified through a review of electronic medical records using diagnostic terms related to "rectal foreign body". A total of 39 patients met the inclusion criteria and were included in the analysis.

Inclusion and exclusion criteria

Patients were included if they were 18 years of age or older and had a confirmed diagnosis of a retained rectal foreign body. Patients were excluded if they presented with rectal or anal trauma without a retained foreign object, such as penetrating injuries, assaults, or instrumentation injuries where no object remained in situ.

Data collection

Data collected included demographic information such as age, gender, and ethnicity, along with the date of hospitalization and the type of rectal foreign body involved. Additionally, the diagnostic methods used, whether rectal examination only or with a rigid rectoscope, and additional imaging modalities such as X-rays and CT scans, were recorded. Details of the extraction procedure, including whether it was performed bedside in the emergency room or required surgical intervention, as well as the post-extraction course and any complications, were documented. Outcome measures included management approach (operative vs non-operative), need for laparotomy, and postoperative complications. Data were retrieved from the institutional electronic medical record and emergency department log system of Rambam Health Care Campus.

Management decision criteria

Management decisions were made according to institutional practice, beginning with the least invasive approach (bedside extraction) and escalating to examination under anesthesia or laparotomy when bedside attempts failed, when objects were not reachable transanally, or when complications such as suspected perforation or peritonitis were present. Postoperative complications were defined as any adverse event occurring during the index admission and were recorded based on documentation in the medical record. Cases with incomplete or missing key clinical data were excluded from the final analysis.

Statistical analysis

Data were imported into Excel (Microsoft, Redmond, Washington) for group allocation, and statistical analyses were conducted using SPSS software v 24.0 (IBM Inc., Armonk, New York). One-way Analysis of Variance (ANOVA) was employed to compare continuous variables between groups, while the Chi-squared test (X²) and Fisher's exact test were used for analyzing categorical variables. A significance level of 0.05 (𝛼) was established for all analyses, with post hoc tests (Tukey Test or Bonferroni Test) performed to discern statistically significant differences between groups.

## Results

A total of 39 patients were included in the study over the 10-year period. The majority were male (74.4%), while females comprised 25.6% (Table 1). The mean age differed significantly between genders, with males presenting at an older age compared to females (49.4 ± 19.3 years vs. 35.4 ± 12.5 years, p=0.015). Most patients were of Jewish ethnicity (87.2%), with Arabs representing 12.8%.

**Table 1:** Patient characteristics Demographic data and foreign-body categories for all patients with anorectal foreign bodies (AFBs). Continuous variables are presented as mean ± standard deviation, and categorical variables as counts and percentages. Comparisons between groups were performed using appropriate statistical tests, with p<0.05 considered significant.

Variable	Male (n=29)	Female (n=10)	Total (n=39)	p-value
Gender				
Male	-	-	29 (74.4%)	-
Female	-	-	10 (25.6%)	-
Age (years), mean ± SD	49.4 ± 19.3	35.4 ± 12.5	45.9 ± 18.5	0.015
Ethnicity	-	-	34 (87.2%) Jewish / 5 (12.8%) Arab	-
Type of foreign body				
Food products, n (%)	12 (30.8%)	2 (5.1%)	14 (35.9%)	0.279
Sex toys, n (%)	5 (12.8%)	5 (12.8%)	10 (25.6%)	0.087
Unspecified objects, n (%)	8 (20.5%)	0 (0%)	8 (20.5%)	0.086
Other, n (%)	4 (10.3%)	3 (7.7%)	7 (18.0%)	0.344

The type of anorectal foreign body varied by gender. Food products were the most frequent objects overall (30.8% in males and 5.1% in females), but this difference was not statistically significant (p=0.279). Sex toys were reported equally among males and females (12.8% each, p=0.087). Unspecified objects were found exclusively among male patients (20.5%), but this difference did not reach statistical significance (p=0.086). Other miscellaneous objects, such as glassware or rods, were seen in 10.3% of males and 7.7% of females (p=0.344). No statistically significant gender differences were observed in the distribution of foreign-body types (Table 1).

With regard to clinical management (Table 2), nearly half of the patients (48.7%) required surgical intervention. The need for surgery varied according to object type, with food products, sex toys, and other solid items requiring surgery in 15.4%, 17.9%, and 12.8% of cases, respectively. None of these differences were statistically significant (p=0.741, p=0.155, and p=0.235, respectively). Manual extraction was successful in 28.2% of patients, and spontaneous expulsion occurred in 7.7%.

**Table 2:** Clinical outcomes Clinical outcomes including operative and non-operative management of patients with anorectal foreign bodies. Data are presented as counts and percentages. Statistical comparisons were performed using Fisher’s exact test due to small subgroup sizes.

Clinical outcome/parameter	Number of patients	Percentage (%)	p-value
Need for surgical intervention			
Surgical intervention required	19	48.70%	
Food products	6	15.40%	0.741
Sex toys	7	17.90%	0.155
Other solid items	5	12.80%	0.235
Unspecified objects	1	2.60%	-
Manual extraction successful	11	28.20%	-
Spontaneous extraction	3	7.70%	-
Manual/spontaneous success			
Food products	4	10.20%	0.729
Sex toys	2	5.10%	0.279
Other Solid Items	2	5.10%	1
Unspecified objects	6	15.40%	0.016

When analyzing the success of non-operative management across object types, removal was most successful in cases of unspecified objects, which showed a statistically significant association with successful manual or spontaneous extraction (p=0.016). For food products (p=0.729), sex toys (p=0.279), and other solid items (p=1.000), no significant differences were observed.

Diagnostic modalities and complications are summarized in Table 3. Most diagnoses were established clinically, with physical examination alone being sufficient in 71.8% of cases, while 28.2% required adjunctive tools such as radiographs, CT imaging, or rigid rectoscopy. Among the 19 patients requiring surgery, the majority underwent examination under anesthesia (EUA, 43.6%), and five patients (12.8%) required exploratory laparotomy, including three cases converted from EUA after failed extraction attempts. Postoperative complications were rare, limited to a single case (2.6%) of surgical-site infection (p=0.128, not statistically significant). No mortality occurred during the study period.

**Table 3:** Diagnostic methods Diagnostic modalities and operative management strategies used in patients with anorectal foreign bodies. Data are presented as counts and percentages.

Diagnostic method	Number of patients	Percentage (%)	p-value
Physical examination only	28	71.80%	-
Additional diagnostic tools used	11	28.20%	-
Type of surgical intervention			
Examination under anesthesia (EUA)	17	43.60%	-
Exploratory laparotomy	5	12.80%	-
Complications post-surgery			
Surgical site infection	1	2.60%	0.128

Ethnicity was recorded as part of routine demographic documentation but was not analyzed as an independent variable due to limited subgroup sizes and the exploratory nature of the study.

Regarding the five cases that required more invasive procedures due to complications or failure of initial extraction attempts; the first patient was a 33-year-old female who presented to the emergency room (ER) after inserting a sex toy into her rectum. Multiple attempts to extract the foreign body in the ER were unsuccessful. The patient was taken to the operating room (OR) for examination and extraction under general anesthesia; however, this attempt also failed. Subsequently, a laparotomy was performed, revealing the foreign body lodged at the splenic flexure of the colon. The object was carefully milked distally into the rectum and successfully extracted through the anus without the need for a colotomy. The patient had an uneventful postoperative recovery and was discharged on postoperative day (POD) four.

The second patient was a 69-year-old male who presented to the ER after inserting a plastic water bottle into his rectum. A computed tomography (CT) scan revealed a foreign body in the colon with free air in the abdominal cavity, consistent with a perforation. The patient underwent an emergency laparotomy, which revealed a tear in the sigmoid colon and a plastic bottle in the peritoneal cavity. The perforation was repaired with primary suturing, and a loop diverting colostomy was created proximal to the repair site. The patient had an uneventful postoperative recovery and was discharged on POD five.

The third patient was a 40-year-old male who presented to the ER after inserting an avocado into his rectum for sexual gratification. Similar to the first case, repeated attempts at extraction in the ER were unsuccessful. The patient was taken to the OR for manual extraction under general anesthesia, but this attempt also failed. A laparotomy was performed, which revealed the avocado lodged in the sigmoid colon. The object was milked distally into the rectum and successfully extracted through the anus. The patient recovered well postoperatively.

The fourth patient was a 71-year-old male who presented to the ER after inserting a cylindrical glass object into his rectum. Repeated attempts at extraction in the ER failed. Colonoscopy revealed the glass object in the sigmoid colon, with the top half broken and glass fragments embedded in the colonic mucosa. Suspicion of perforation led to a laparotomy, which confirmed a sigmoid colon perforation with minimal spillage. A colotomy was performed to remove the intact portion of the glass object and extract all glass fragments. The colotomy was sutured primarily. The patient developed a superficial wound infection during the postoperative period, which was treated appropriately. He recovered without further complications.

The fifth patient was a 55-year-old male who presented to the ER after inserting a large cucumber into his rectum. Initial bedside attempts at removal failed, and the patient was taken to the OR for examination under anesthesia. The object could not be retrieved transanally, and a laparotomy was performed. The cucumber was identified in the sigmoid colon, and gentle milking into the rectum allowed successful transanal extraction without colotomy. The patient had an uncomplicated postoperative course and was discharged on POD three.

## Discussion

This series provides a 10-year experience with anorectal foreign bodies (AFBs) at a tertiary referral center, demonstrating important demographic, clinical, and management trends. The majority of patients were male, which aligns with previously published data showing that AFBs are more commonly encountered in men [3]. However, our study also revealed a significant age difference, with males presenting later in life than females (p=0.015). This observation may suggest different psychosocial or behavioral factors influencing presentation patterns between the sexes. 

The types of foreign bodies in our series were diverse, consistent with previous literature documenting a wide range of objects, including household items, bottles, light bulbs, and sexual devices [1,2]. In our cohort, food products were the most common, echoing earlier findings that such objects are frequently used in self-insertion [2,3]. Sex toys were equally represented in both genders, with no statistically significant difference between male and female patients (p=0.087). This contrasts with earlier reports describing sex-toy use as predominantly male [2,3], potentially reflecting cultural differences or evolving sexual practices in our population. Unspecified objects were found exclusively in male patients, although this difference did not reach statistical significance (p=0.086). This may reflect reluctance to disclose the inserted object, a phenomenon also highlighted in prior studies where under-reporting due to embarrassment is common [3]. Given the small subgroup sizes, analyses should be interpreted as exploratory.

Almost half of our patients required surgical intervention (48.7%), a higher proportion compared with some series where bedside removal or transanal approaches predominate [6]. This may reflect delayed presentation or referral bias to our tertiary institution. Non-operative management was successful in roughly one-third of patients, with spontaneous passage occurring in 7.7%. Importantly, unspecified objects were significantly more likely to be successfully managed non-operatively (p=0.016), possibly reflecting object characteristics such as size, shape, or material that facilitated removal.

Complication rates were low, limited to a single case (2.6%) of surgical-site infection occurring in a laparotomy patient (p = 0.128, Fisher's exact test, not statistically significant). This aligns with prior data showing that while complications such as bleeding, perforation, obstruction, and sepsis can occur, overall morbidity and mortality remain low when patients are managed promptly [5-7]. Nonetheless, the need for laparotomy in over 12% of patients underscores the potential seriousness of these cases and the resources required for their management.

Our diagnostic approach reflects current practice, with over 70% of patients managed based on clinical examination alone and imaging reserved for complex or complicated cases [4,8]. This is in keeping with published recommendations emphasizing careful history, digital rectal examination, and selective imaging [4,8].

Treatment strategy depends on the patient's condition and the type, size, and location of the foreign body. Management should progress from the least to the most invasive technique, beginning with manual extraction under sedation or anesthesia [8]. If the object is beyond the peritoneal reflection and not safely retrievable transanally, proctosigmoidoscopy with snares, forceps, or baskets may be used [9]. When these fail, examination under anesthesia can facilitate relaxation and transanal extraction [8]. In cases of perforation, obstruction, or failed less-invasive attempts, laparotomy may be necessary. Intra-operative options include milking the object distally, performing a colotomy, or a Hartmann procedure if peritonitis is present. Laparoscopic approaches may also be considered in selected patients [10-13]. Vigilance for peritonitis remains essential, as its presence necessitates urgent surgical exploration to prevent severe complications [12]. Post-extraction sigmoidoscopy is recommended to assess for mucosal injury or bleeding, which may not be clinically apparent [14].

This study has several limitations. Its retrospective design introduces potential bias, and the modest sample size limits statistical power. Under-reporting due to stigma likely results in underestimation of true incidence [3]. Nevertheless, this series represents one of the largest single-center reports from our region and provides meaningful insights into both clinical patterns and outcomes. Future work should aim to identify predictors for surgical intervention, which could aid in patient triage and optimize management. Public awareness, sexual health counseling, and stigma reduction may also help prevent risky behaviors leading to AFB insertion [3].

Additionally, management decisions were based on clinician judgment rather than a standardized institutional protocol, which may introduce inter-operator variability in treatment selection. Long-term follow-up data were not consistently available due to the retrospective nature of the study, limiting assessment of delayed complications or recurrence.

## Conclusions

This 10-year experience with anorectal foreign bodies at a tertiary referral center highlights the diversity of presentations and the complexity of management. Although the condition remains predominantly seen in males, a significant age difference between genders was observed, whereas the distribution of object types did not differ significantly. Nearly half of the patients required surgery, with a minority undergoing laparotomy due to complications or failed transanal extraction. Despite the potential for serious outcomes, complication rates in this series were low, reflecting the effectiveness of timely diagnosis and a structured, stepwise management strategy.

Management should progress from the least to the most invasive approach, beginning with bedside or manual extraction, advancing to endoscopic or examination under anesthesia, and reserving laparotomy for refractory or complicated cases. Post-extraction sigmoidoscopy is advisable to identify mucosal injuries or bleeding.

These findings emphasize the importance of early recognition, appropriate triage, and surgical preparedness in the management of anorectal foreign bodies. Future efforts should focus on preventive strategies, including sexual health education and stigma reduction, which may ultimately lower incidence and improve outcomes. Stigma reduction and early recognition may represent potential areas for future study and prevention efforts.
